# Mucosal immune response modulated by secreted and membrane-bound hydrolases of *Candida albicans* in vulvovaginal candidiasis

**DOI:** 10.3389/ffunb.2025.1692795

**Published:** 2025-12-11

**Authors:** Guocheng Qian, Li Ding, Chao Tan, Lidan Wang, Cong Long

**Affiliations:** Department of Clinical Laboratory, Jingjiang People’s Hospital Affiliated to Yangzhou University, Taizhou, Jiangsu, China

**Keywords:** *Candida albicans*, mucosal immunity, VVC, hydrolases, therapy

## Abstract

Vulvovaginal candidiasis (VVC) affects the physical and mental health of millions of women worldwide. The leading cause of VVC, *Candida albicans*, can induce a strong mucosal inflammatory reaction during the VVC infection, where secreted and membrane-bound adhesion and hydrolases seem to be the key virulent factors to promote the mucosal antifungal immunity and immunopathology. Several hydrolases, such as Saps, Als, candidalysin, lipases, and phospholipases, have been identified in vaginal secretions isolated from VVC patients; however, the immune impacts of some hydrolases have not been well documented. In this review, we focus on the literature that addresses the immunopathogenic roles of the Als adhesin family or proteinase, such as Sap and candidalysin, in VVC. Our goal is to expand our knowledge of VVC pathogenesis in order to provide new strategies for VVC treatment.

## 1 Introduction

Mucosal inflammatory reactions in vulvovaginal candidiasis (VVC) often present with clinical complaints of excess vaginal discharge, vulvar erythema, swelling, and pain (Maraki et al., 2019). The prevalence of VVC by age 50 varies widely in different regions, and 75% of women would experience it at least once in their lives (Mtibaa et al., 2017). Nearly half of patients with vaginal fungal infection even experience three or more episodes of infection within a year—a condition defined as recurrent vulvovaginal candidiasis (RVVC). The latter can seriously affect quality of life, mental health, and sexual activity in these patients (Amouri et al., 2011). The genus *Candida* remains as the most common fungal pathogen in humans, accounting for 94.8% of vaginal fungal infections (Achkar and Fries, 2010), and the majority of *Candida* isolates are *C. albicans* (Jafarzadeh et al., 2022).

**Figure 1:**
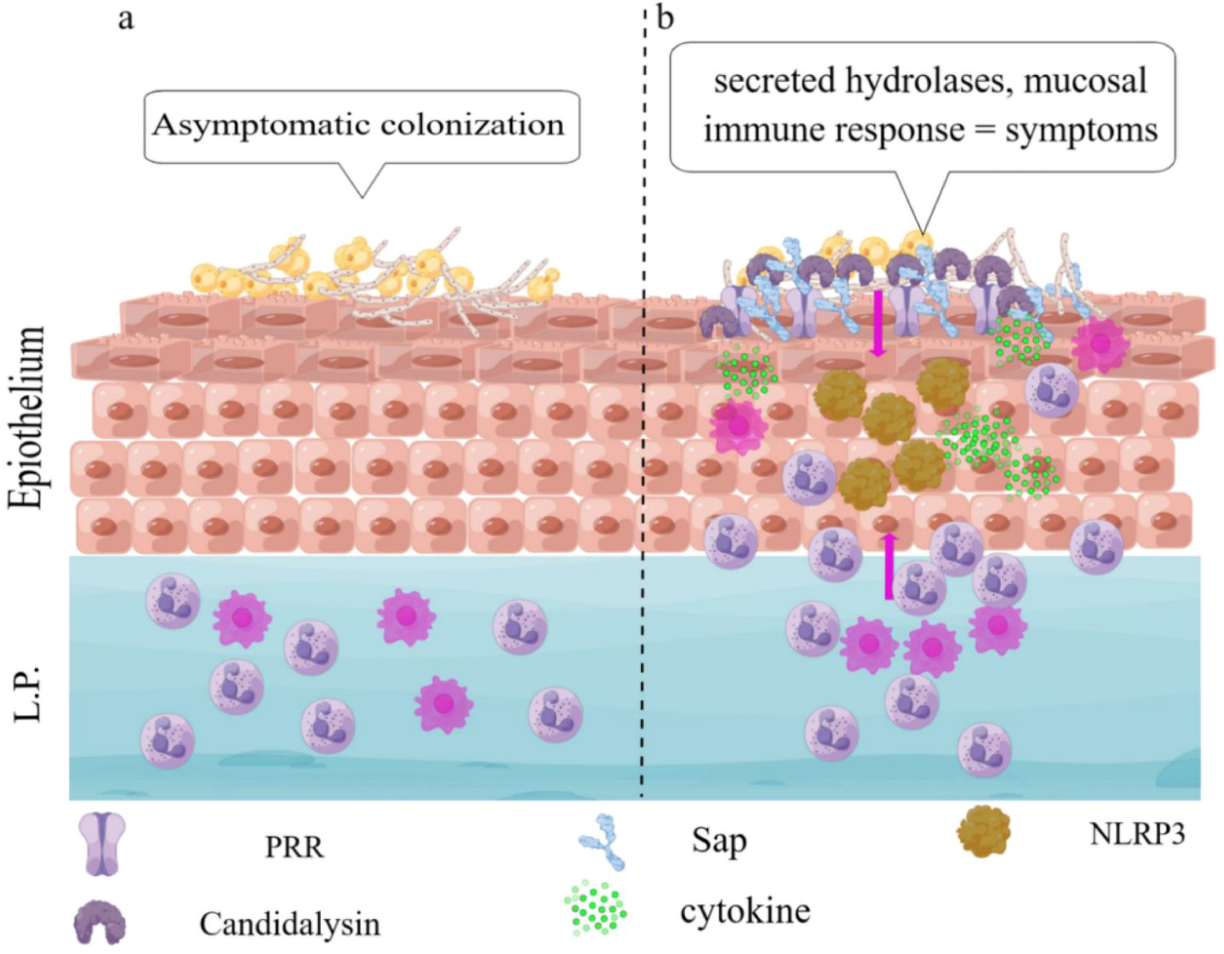
An updated working model of the immunopathogenesis of *C*. *albicans* vaginitis. **(A)** Yeast and hyphal forms of *C*. *albicans* asymptomatically colonize the vaginal epithelium. **(B)** Mucosal immune response modulated by secreted and membrane-bound hydrolases of *C. albicans* results in VVC.

Hyphae formation has long been considered an invasive trait of *C. albicans*, which normally inhabits the mucosa in its yeast form under physiological conditions. The mechanical penetration of vaginal epithelial cells (ECs) by hyphae is more prone to induce mucosal immunity of the host (Gonçalves et al., 2016; Czechowicz et al., 2022). However, a study has shown that *C. albicans* hyphae lacking hydrolases such as ECE1 and SAP show a reduction or complete loss of their ability to cause VVC (Consolaro et al., 2005), suggesting the importance of these hydrolases on VVC pathogenesis. Some hydrolases are secreted by the hyphal form of *C. albicans* that activate vaginal EC to release a variety of inflammatory factors (such as IL-1 and IL-18) for the development of VVC. The activated EC and cytokines then recruit neutrophils to further aggravate the inflammatory response in local areas (Roselletti et al., 2017). In this review, we summarize the effects of *C. albicans* hydrolases on mucosal immunity and their potential pathogenic mechanisms in VVC. Our goal is to find new therapeutic strategies for the improved treatment and prevention of VVC and RVVC (**Figure 1**).

## 2 Secreted aspartyl proteases

Saps (secreted aspartyl proteases) include a group of proteases that help fungal cells hydrolyze host tissues during invasiveness (Pericolini et al., 2015; Cauchie et al., 2017). In *C. albicans*, Saps are encoded by the *SAP* genes *SAP1–10* (Kumar et al., 2022), of which Sap1–3 are primarily secreted by the yeast form of *C. albicans* and are mainly involved in early mucosal immune responses (Maia et al., 2021; Peng and Tang, 2021). Sap4–6, produced by the hyphal form of *C. albicans*, are mainly associated with mucosal invasion, and Sap9–10 are related to *C. albicans* cell wall composition (Willems et al., 2017). The substrates of these Sap proteases are either protein constituents of ECs or proteins with antimicrobial activity (Lim et al., 2021). It has been known that protease activities of these Saps are maximized when mucosal pH is low (pH < 4) (Cauchie et al., 2017). Sap3 has long been recognized as a key molecule aiding in the adaptation of *C. albicans* to acidic environments (De Bernardis et al., 2018), and the immunopathologic effects of Saps on VVC seem to be more related to the ones produced in the hyphal form of growth (Willems et al., 2017). A study has shown that the expression levels and activity of Saps are higher in VVC women than in those who are asymptomatic vaginal *Candida* carriers (Gonçalves et al., 2016). While the expression of each *SAP* gene varies according to the vaginal secretions of VVC patients, *SAP2* is detectable in almost all patients with VVC, followed by *SAP6* (Roselletti et al., 2017). Today, high expressions of Sap2 and Sap6 have been used as indicators of inflammation in VVC (Pericolini et al., 2015; Gabrielli et al., 2016; Roselletti et al., 2017).

Saps are involved in the pathogenesis of VVC through activation of the inflammatory signaling pathways in vaginal EC, such as the NLRP3 inflammasome pathway (Rodríguez-Cerdeira et al., 2019). For example, Sap2 activates the NLRP3 inflammatory pathway, which leads to the activation of caspase-1 and subsequently promotes the release of IL-1β and IL-18 (Roselletti et al., 2017). A recent study has shown that Sap6 produced by *C. albicans* hyphae can initiate oral mucosal immunity via the protease-activated receptor (PAR2) expressed on the cell surfaces of ECs (Kumar et al., 2022). IL-1β and IL-18 are the main pro-inflammatory molecules in VVC since their expression levels in vaginal secretions are associated with clinical symptoms of patients with VVC (Roselletti et al., 2017). The promotion of inflammation led by IL-18 is due to a downregulation of IL-22 response that normally inhibits NLRP3 inflammatory activation in VVC (Borghi et al., 2019). The link for Sap2 or Sap6 on this inflammasome pathway is caspase-11 activation through the type I interferon (IFN) pathways (Gabrielli et al., 2015); when synergized with caspase-1, this caspase-11 activation can greatly increase IL-1β production (Pietrella et al., 2013). In addition, Saps also play important enzymatic roles to effectively disrupt antimicrobial and immunomodulatory properties, such as IL-37, a member of the IL-1 cytokine family, produced in the vaginal mucosa (Rapala-Kozik et al., 2015). Although the roles of IL-37 in VVC mucosal immunity have not been confirmed, IL-37 could inhibit the activation of caspase-11, which is the target of IL-1β activation.

**Figure 2:**
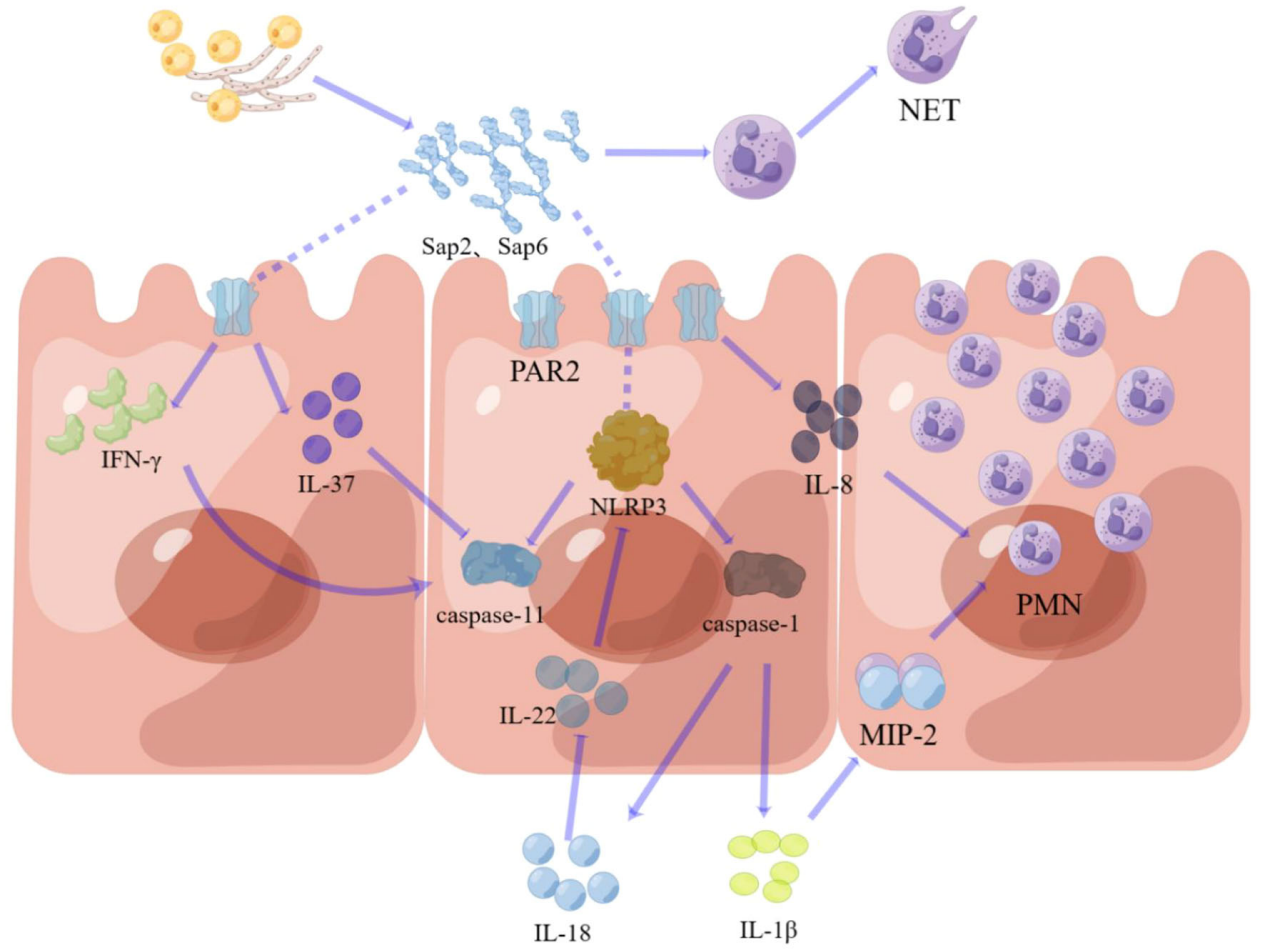
The mucosal immune response triggered by **Saps. Receptor recognition:** Saps initiate signaling by activating protease-activated receptor 2 (PAR2) on vaginal epithelial cells. **Signal transduction pathways:** Saps activate dual pro-inflammatory pathways: the NLRP3 inflammasome, which activates caspase-1, and the type I interferon pathway, which induces caspase-11. These caspases synergistically amplify the inflammatory signal. **Effector molecule functions:** The resulting response includes: neutrophil recruitment driven by the IL-1β -MIP-2 axis and IL-8; sustained inflammation mediated by IL-18 via suppression of IL-22; and amplification of inflammation due to Sap-mediated degradation of the anti-inflammatory cytokine IL-37.

Furthermore, Sap2 and Sap6 play important roles in the recruitment and activation of neutrophils in the vaginal mucosa during infection (Pericolini et al., 2015). Although Sap-stimulated IL-1β production can directly stimulate neutrophil recruitment through the MIP-2 (macrophage inflammatory protein-2) mechanism (Gabrielli et al., 2016), neutrophil recruitment in the vagina also occurs in response to an increase in the chemokine IL-8 (Gabrielli et al., 2016). Neutrophil extracellular traps (NETs) have been recently recognized as an important mucosal immune response to invasive *C. albicans* (He et al., 2022). However, few studies have investigated the mechanism of Sap-mediated NETs in the immunopathology of VVC. In addition to the IL-8-induced neutrophil chemotaxis, Saps also degrade AIPI (α1-proteinase inhibitor) to promote neutrophil extracellular trap formation (Gogol et al., 2016). New evidence has shown that Sap-induced neutrophil extracellular traps interact with CD11b/CD18, CD16, and CD14 receptors (He et al., 2022) (**Figure 2**).

## 3 Candidalysin

Candidalysin was the first discovered toxin of *C. albicans* that is encoded by *ECE1* (extent of cell elongation 1) (Naglik et al., 2019). The absence of the *ECE1* gene prevents *C. albicans* from damaging the vaginal mucosal barrier (Moyes et al., 2016; Naglik et al., 2019) and significantly reduces its pathogenicity in VVC (Liu et al., 2021). The loss of the ability to invade the mucosal barrier demonstrates the importance of candidalysin in mucosal inflammation caused by *C. albicans.*

The invasive hyphae of *C. albicans* form an invasive pocket that is a prerequisite for candidalysin-mediated mucosal immunity (Mogavero et al., 2021). Upon hyphae formation, candidalysin can activate epithelial cell inflammation via EGFR binding (Ho et al., 2019) and, consequently, MAPK and p38 activation for inflammatory response (Zhang et al., 2022). In addition, candidalysin can stimulate the NLRP3 inflammatory pathway through increased potassium outflow and calcium inward flow in vaginal EC (Rogiers et al., 2019). Excessive NLRP3 responses, accompanied by the continuous production of IL-1β, then give rise to a major feature of VVC pathogenesis (Zhang et al., 2022). Evidence from studies has shown that candidalysin can stimulate vaginal EC to produce immune effector molecules, including IL-1α, IL-1β, G-CSF, GM-CSF, IL-6, and IL-8 (Richardson et al., 2018). Among these, IL-1α can initiate an immune response in the oral mucosa via the IL-1 receptor, resulting in the release of GM-CSF (granulocyte-macrophage colony-stimulating factor) and IL-8 (Hanaoka and Domae, 2021). GM-CSF and IL-8 then recruit neutrophils to amplify inflammation in the mucosa. Although IL-6 has been proven to trigger an inflammatory response in the gut (Wu et al., 2022), there is still no evidence to show the role of IL-6 in the pathogenesis of VVC.

In addition to its direct effects on ECs, candidalysin has been reported to activate a variety of innate immune effector cells, including neutrophils, macrophages, and mast cells. In VVC pathology, neutrophil recruitment seems not to be critical for *C. albicans* clearance (Ho et al., 2019), but the increased neutrophils can exacerbate the inflammatory response in the vagina (Yano et al., 2018). Candidalysin promotes the expression of neutrophil chemotactic factors such as CXCL2 and CXCL3 in vaginal ECs (Richardson et al., 2018), and *C. albicans* lacking the *ECE1* gene results in a significant reduction in neutrophils (Richardson et al., 2018). When compared with neutrophils, macrophages can kill *C. albicans* to some extent in the vagina; meanwhile, they produce other inflammatory factors for inflammation in the vagina (Bojang et al., 2021). For example, candidalysin can promote the production of Gasdermin D (GSDMD) pores that would disrupt macrophage membranes or activate the NLRP3-mediated IL-1β inflammatory pathway (Kasper et al., 2018; König et al., 2020; Ding et al., 2021).

**Figure 3:**
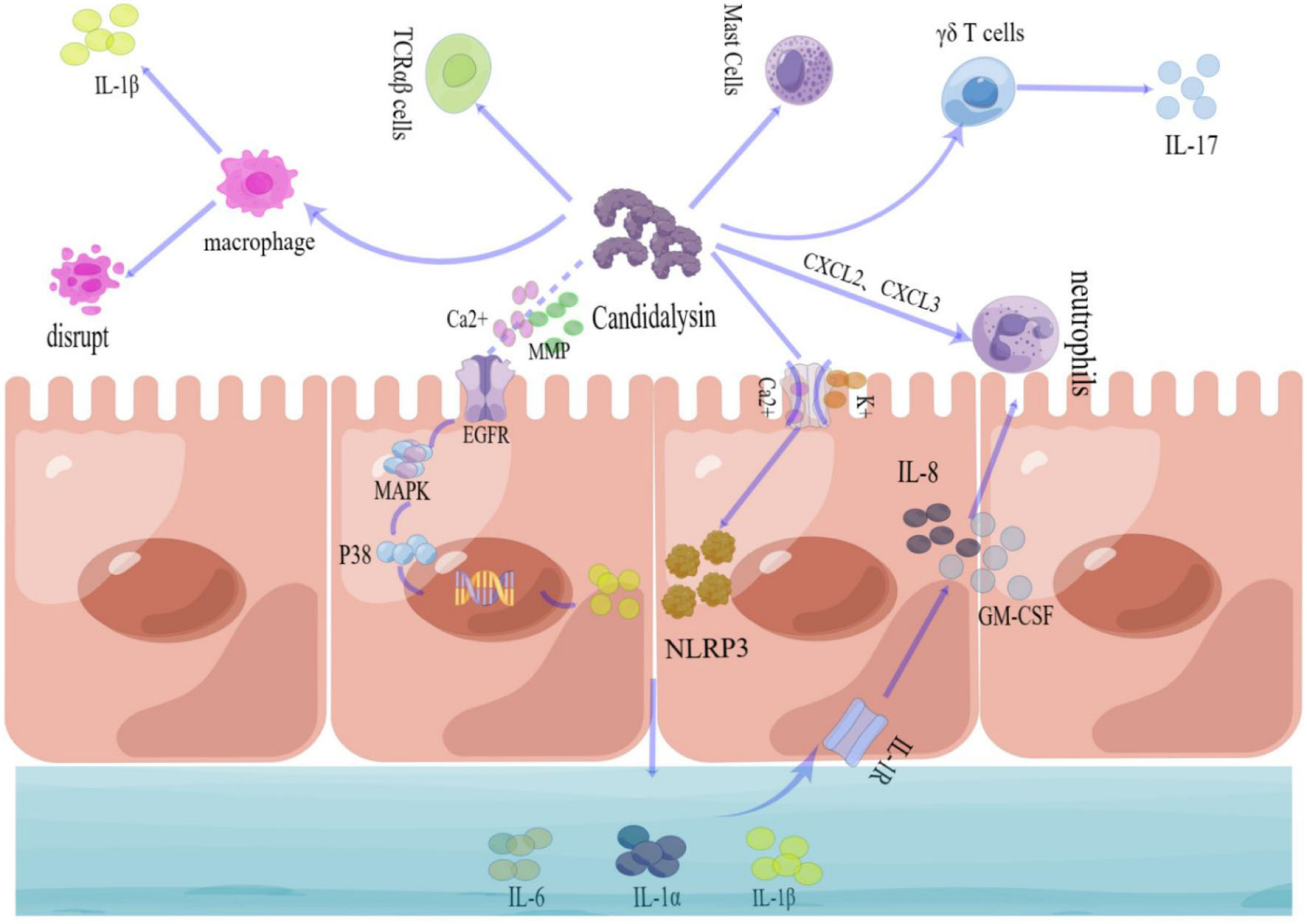
The mucosal immune response triggered by candidalysin. **Receptor recognition:** Candidalysin activates epithelial cells (ECs) via the epidermal growth factor receptor (EGFR). **Signal transduction pathways:** Candidalysin triggers MAPK/p38 signaling and induces calcium (Ca^2+^) influx and potassium efflux to activate the NLRP3 inflammasome in EC. **Effector molecule functions:** IL-1α activates IL-8 and GM-CSF production through the IL-1 receptor, leading to neutrophil recruitment. IL-6 can amplify inflammatory responses in EC. CXCL2 and CXCL3 recruit neutrophils in EC.

Other candidalysin-related effector cells include mast cells and T cells. Mast cells can be stimulated by candidalysin through the Dectin-1 receptor and the MAPK signaling pathways; however, it is not clear what roles mast cells have in the pathogenesis of VVC. Candidalysin stimulates γδ T cells to generate a protective immune response by producing IL-17, a critical cytokine for *C. albicans* clearance in the mucosa (Peters et al., 2020; Willems et al., 2020). Candidalydin enables the amplification of innate TCRαβ cells to trigger an innate alarm (Li et al., 2017). Nevertheless, the two key functions of candidalydin are recruiting neutrophils and activation of the NLRP3–IL-1β inflammatory pathway, which undoubtedly prove its roles as virulence factors of *C. albicans* (**Figure 3**).

## 4 Lipases

Lipase, a class of esterase, specifically catalyzes the hydrolysis of triacylglycerol into glycerol and free fatty acid at the oil–water interface (Pellon et al., 2020). Ten lipases (Lip1–10) have been isolated and identified from *Candida*, among which Lip5, 6, 8, and 9 were found to be expressed on *Candida* and secreted during infection (Zhuang et al., 2022). The pathological roles of lipase have been demonstrated by the fact that the fungal load and the inflammatory response will be decreased if the mucosal surface is infected with *Candida* lacking lipase (Shao et al., 2022). Mechanically, lipases induce inflammation via activating macrophages that produce excessive ROS to damage the mucosal barrier (Paraje et al., 2009). However, based on the data that a higher enzymatic activity is found in the vaginal secretions of VVC, lipases may also participate in the inflammatory response in VVC via promotion of cytokine and chemokine secretion in ECs and immune cells (Ellepola et al., 2014).

## 5 Phospholipases

Phospholipases (phospholipases A, B, C, and D) catalyze the hydrolysis of phospholipids, which aid in degrading mucosal cells for fungi to penetrate the surface barrier. Phospholipase B1, as the main virulence factor, plays a key role in the invasion of *Candida* spp. and induces an inflammatory response (Richardson et al., 2019). A study has shown that phospholipase can lead to an increased risk of mucosal infections (Ramla et al., 2016), while inhibition of phospholipase activity can lead to clinical antifungal effects (Ellepola et al., 2014). Phospholipase activity is found highest in *C. albicans*, followed by *C. tropicalis*, which is consistent with the population of isolated *Candida* spp. in the vagina. However, drugs targeting the inhibition of phospholipase have not been developed for the treatment of VVC.

## 6 Agglutinin-like sequence

Adhesin proteins aid *C. albicans* to adhere to the mucosal epithelium for colonization (Cota and Hoyer, 2015) and are mostly produced in hyphal form of growth, such as the Hwp1 (hyphal wall protein) and Als (agglutinin-like sequence) families. The Als family includes a group of glycoproteins that are found on the surface of *C. albicans*, encoded by the *ALS* genes (*ALS1–7* and *ALS9*) (Monroy-Pérez et al., 2012). The expression of *ALS* genes is upregulated in vaginal secretions of VVC patients (Bonfim-Mendonça et al., 2021), and *C. albicans* isolates from patients with VVC showed greater adherence to vaginal EC than those from asymptomatic *C. albicans* carriers *in vitro* (Bonfim-Mendonça et al., 2021).

However, different adhesin proteins may act differently when associated with mucosal immunity (Sui et al., 2017). For example, the binding of Als3 with cadherins, EGFR (epidermal growth factor receptor), or HER2 (human epithelial receptor 2) on EC can initiate *C. albicans* endocytosis (Branco et al., 2021). The cell membrane receptor EGFR can be activated by all pathogens, including fungi, triggering the mucosal inflammatory processes (Pastore et al., 2014; Mukherjee and Balaji, 2019). Endocytosis by vaginal mucosal cells is also the invasion step of *C. albicans* to trigger VVC (Zhang et al., 2018). Upon endocytosis, intracellular vesicles are formed in ECs, in which *C. albicans* toxins are accumulated that would stimulate the immune response in the mucosa (Swidergall et al., 2021). Furthermore, mucosal NK cells also contribute to mucosal antifungal immunity through cytolytic function and IFN-γ production (Ivanova et al., 2014). Interestingly, Als proteins bind to a checkpoint receptor TIGIT (T-cell immunoreceptor with Ig and ITIM domains) on NK cells, which then triggers this cytolytic function of NK cells for *C. albicans* clearance (Charpak-Amikam et al., 2022).

## 7 Conclusion and perspective

VVC has been treated as a neglected disease despite having a high incidence. The presence of hyphae in vaginal secretions has been a marker of *Candida* invasion in VVC diagnosis. The secreted and membrane-bound hydrolases of *Candida* that are associated with hyphal formation interact with inflammatory pathways in ECs and innate immune cells to maintain a healthy fungal–host relation. The disruption of intravaginal homeostasis due to vaginal microbiota dysbiosis or hormonal fluctuations upregulates the activity and expression of *Candida*-secreted and membrane-bound hydrolases, subsequently triggering a predominant immune response involving epithelial cells and innate immune cells that ultimately leads to the onset of VVC symptoms.

Azole antifungals, as the conventional therapy for VVC, are limited by their inability to resolve clinical symptoms and their propensity to induce drug resistance. Drugs targeting *Candida* hydrolases can effectively circumvent the emergence of drug resistance, while the topical application of inflammatory cytokine-neutralizing agents can alleviate associated inflammatory symptoms. Artificial Sap inhibitors targeting Sap2 were developed some time ago (Cadicamo et al., 2013). More recent findings show that specific imidazole-based compounds and plant extracts also inhibit Sap activity to impart antifungal effects (Chakraborty et al., 2023). Additionally, research in systemic *Candida* infection models demonstrates that albumin effectively mitigates candidalysin-induced epithelial damage. In addition, multiple strategies for neutralizing IL-1β have been developed, such as the IL-1 receptor antagonist (IL-1Ra) and anti-IL-1β monoclonal antibodies. IL-1β neutralizers have been reported to be applied in the treatment of various acute and chronic inflammatory conditions (Dinarello, 2004). More recently, anti-IL-18 monoclonal antibodies have also been developed to treat IL-18-mediated inflammatory responses (Li et al., 2021). However, for both types of monoclonal antibodies, topical formulations—such as the development of corresponding gels—represent a promising yet unreported direction for VVC treatment. Lastly, the latest research demonstrates that high-dose phototherapy effectively eradicates *Candida* biofilms and mitigates vaginal epithelial injury. This effect becomes markedly more pronounced when enhanced by a nanomaterial platform, suggesting considerable potential for clinical VVC management (Lin et al., 2023).
